# Thermodynamical journey in plant biology

**DOI:** 10.3389/fpls.2015.00481

**Published:** 2015-06-30

**Authors:** Adelin Barbacci, Vincent Magnenet, Marc Lahaye

**Affiliations:** ^1^Biopolymers Interactions Assembly, Institut National de la Recherche Agronomique, UR 1268Nantes, France; ^2^Laboratoire des Interactions Plantes Microorganismes, Institut National de la Recherche Agronomique, UMR441Castanet-Tolosan, France; ^3^Laboratoire des Interactions Plantes Microorganismes, Centre Nationale de la Recherche Scientifique, UMR2594Castanet-Tolosan, France; ^4^Laboratoire des Sciences de l'Ingénieur, de l'Informatique et de l'Imagerie (ICube), Université de Strasbourg, UMR Centre National de la Recherche Scientifique 7357Illkirch, France

**Keywords:** integrative biology, plant sciences, nonequilibrium thermodynamics, dissipative structure, modeling

## Abstract

Nonequilibrium irreversible thermodynamics constitute a meaningful point of view suitable to explore life with a rich paradigm. This analytical framework can be used to span the gap from molecular processes to plant function and shows great promise to create a holistic description of life. Since living organisms dissipate energy, exchange entropy and matter with their environment, they can be assimilated to dissipative structures. This concept inherited from nonequilibrium thermodynamics has four properties which defines a scale independent framework suitable to provide a simpler and more comprehensive view of the highly complex plant biology. According to this approach, a biological function is modeled as a cascade of dissipative structures. Each dissipative structure, corresponds to a biological process, which is initiated by the amplification of a fluctuation. Evolution of the process leads to the breakage of the system symmetry and to the export of entropy. Exporting entropy to the surrounding environment corresponds to collecting information about it. Biological actors which break the symmetry of the system and which store information are by consequence, key actors on which experiments and data analysis focus most. This paper aims at illustrating properties of dissipative structure through familiar examples and thus initiating the dialogue between nonequilibrium thermodynamics and plant biology.

“Thermodynamics is the only physical theory of universal content which, within the framework of the applicability of its basic concepts, I am convinced will never be overthrown.”

Albert Einstein

## 1. Introduction

Physics and biology share a common history. Lamarck stated in 1870 “life is nothing else than a physical phenomenon” (de Lamarck and Dalloz-Bourguignon, 1988). Thus, from the pioneering work of Darwin and Darwin (1880) on plant movement to the structure of DNA (Watson and Crick, 1953), advances in biology were based on physics. With time, technical advances in genetics made it possible to focus on the identification and the characterisation of single molecules and therefore to promote more and more reductionist approaches. During the last few decades, successes of molecular approaches led to the belief that biology could be reduced to genetics. However, the exponential growth of molecular data available did not allow us to unmask the *big picture* by offering a holistic biology.

Physics propose an elegant alternative to provide a simpler view of the complexity (Wolgemuth, 2011) by emphasizing underlying mechanisms which link genes expression to plant functions (Zwieniecki and Dumais, 2011) and thus offering biology fundamentals explanations (Knight, 2002).

This domain is split into several branches which all share energy as common physical quantity. Life is also governed by energy and its transfers as for instance, the conversion of light energy in chemical energy through photosynthesis and the Calvin's cycle. The science studying the transfers of energy, called thermodynamics, is therefore relevant to be developed in biophysical approaches.

This paper aims at illustrating the intrinsic physical concepts of nonequilibrium thermodynamics through plant biology. This mini-review focuses specifically on the useful concept of dissipative structure (DS) which provides a powerful framework to model life. These concepts can obviously be used to design quantitative approaches. Importance of quantification for biology is discussed in Zwieniecki and Dumais (2011).

## 2. Thermodynamics at equilibrium

**Figure 1:**
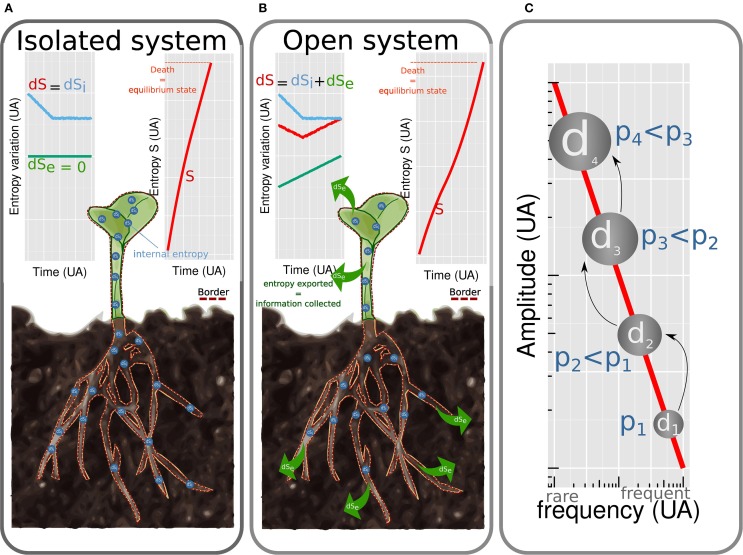
**Plants considered as isolated (A) and open (B) systems**. (*dS* = *dS_i_* + *dS_e_*). In isolated systems no entropy is exchanged (*dS_e_* = 0) with environment. Entropy of the system *S* increases rapidly (*dS* = *dS_i_* > 0). The system tends toward equilibrium, which corresponds to death. For open systems, energy is dissipated and entropy is exchanged with the environment (*dS_e_* < 0). The amount of entropy exchanged is of the same magnitude than the internal entropy produced (*dS_i_* >~ |*dS_e_*|) leading to a slow increase of internal entropy (*S*). Equilibrium state (i.e., death) is reached after a longer time than for isolated systems. Cascade of dissipative structures **(C)**. Amplitude of energy dissipated by a dissipative structure is linked to frequency by an 1/f law.

The first formulation of thermodynamics was developed by Carnot (1824). The ensuing thermodynamics is based on two laws: energy is conserved (first law) and energy is dissipated irreversibly as heat (second law). It refers to isolated systems at equilibrium state. By *system* is meant a portion of the universe isolated mentally from the rest of the universe (Figure 1 border). Isolated systems do not exchange energy nor matter with their environment (Figure 1A). The equilibrium state is characterized by the absence of gradient leading to the vanishing of fluxes and of generalized thermodynamics forces. A few years after Carnot, Clausius (1850) introduced one of the most debated concept in physics: entropy (noted *S* thereafter). This quantity is a measure of energy dissipated as heat and refers to the irreversibility of a phenomenon. When heat is produced, entropy increases in the system until the equilibrium is reached. This simple definition of entropy, central to the analytical formulation of thermodynamics, can be completed by richer notions. These are context dependent (Prigogine, 1989) and the matching between entropy and information will be introduced later. The thermodynamics of isolated systems has been widely used to describe processes involved in plant biology (Wolfe, 2015) such as passive water flow driven by osmosis (Kamiya and Tazawa, 1956; Tyree, 1969), ions fluxes as a function of membrane potential, enzymatic catalysis, regulation of leaf transpiration (Laisk and Walker, 1989), hydronastic rolling of grass leaf (Moulia, 2000), cell expansive growth (Veytsman and Cosgrove, 1998) and obviously DNA conformation (Sugimoto, 2014). Such approaches allowed to understand mechanisms in a single process and determined fundamental variables. However, to shift from the complexity of one process to the complexity of the whole plant, the assumption of closed or isolated system is too restrictive.

Indeed, living organisms exchange energy and matter with their environment (Figure 1B). As noted by Schroedinger (1944), isolated systems tend toward *disordered* states, (molecules are mixed and gradient vanishes) which does not apply to living systems. The latter are, by contrast, characterized by structured organization which varies in time and space.

## 3. Thermodynamics out of equilibrium

Structured organization of living systems is compatible with the second law when considering living organisms as open systems, dissipating energy and exchanging entropy and matter with their environment. For such systems, the variation of entropy *dS* is the sum of the amount of internal entropy created *dS_i_* with the amount of entropy exchanged *dS_e_* < 0, *dS* = *dS_i_* + *dS_e_*. Life is possible on a period of time during which the entropy increases slowly which is true when internal entropy barely exceeds exported entropy *dS_i_* >~ |*dS_e_*| (Figure 1B). The ability to export entropy to the surrounding environment is the core mechanism coordinating homeostasis of living organisms. This thermodynamic reformulation of homeostasis (Henning and Scarfe, 2013) extends the definition proposed by Bernard (1865) who defined homeostasis as a *dynamic equilibrium* needed to ensure life.

Ilya Prigogine (1917–2003), father of nonequilibrium thermodynamics, named such open systems *dissipative structures* (Nicolis and Prigogine, 1977). These structures are associated with the notion of fluxes and exist only when energy is provided to the system. Prigogine demonstrated that DS self-organized by dissipating energy. Thus, irreversible nonequilibrium thermodynamics lead to another paradigm, powerful enough to study the complexity of life. This framework allows joining different scientific fields to place life in different perspectives (Lineweaver and Egan, 2008). Therefore, physicists rapidly described life through irreversible thermodynamics out of equilibrium (Kedem, 1958; Tyree, 1969; Segel and Jackson, 1972). Nonequilibrium thermodynamics was received with enthusiasm by biologists and especially ones who were interested by the central problem of water and nutrient transport in plant. Dainty (1976) wrote that “the only correct theory is that based on the theory of irreversible thermodynamics.” The enthusiasm was raised because nonequilibrium thermodynamics was able to challenge easily the central problem of coupling effects (Tisza et al., 1956) between water and membranes. Within this view, flux of matter and driving force are linked together by conductance coefficients, expressed from Tisza's matrix (Tisza et al., 1956; Magnenet et al., 2012) to model membrane properties. Sum of products of each flux with its associated force describes the rate of entropy i.e., the dissipation function (Zimmermann and Steudle, 1979; Callen, 1985). Nonequilibrium thermodynamics spread to the present days (Le Deunff and Malagoli, 2014) in many fields of the present biology such as ecology (Schneider and Kay, 1994), evolution (Demetrius, 2000), biochemistry (Qian and Beard, 2005), physiology (Toussaint and Schneider, 1998), cell growth (Barbacci et al., 2013). However, one of the main current challenges in plant biology aims at designing a holistic quantitative biology (Westerhoff and Palsson, 2004; Zwieniecki and Dumais, 2011) called systems biology. Systems biology is based on modeling approaches. The most encountered framework is based on a steady state description and has the ability of quantitative prediction for isolated networks (Orth et al., 2010). The multiplication of high throughput methods provides quantitative data relative to large scale networks. Therefore, the actual challenge for system biology shifts toward the modeling of larger scale metabolic networks. The actual framework does not render complexity of couplings between networks and do not capture richness of life. On the contrary, one may surmise that nonequilibrium thermodynamics would be a reasonable tool to tackle this problem (Soh and Hatzimanikatis, 2010).

Per Bak et al. (1988) demonstrated that DS self-organized such as continuous phase transitions. Properties of continuous phase transitions are the same than mathematical objects called bifurcations. Bifurcations are defined by four properties: amplification of fluctuations, breaking of symmetry, creation and storage of information, invariance to scale. DS leads to the apparition of other DS creating a cascade through space and time scale. This phenomenon is commonly called the *butterfly effect*. Whereas isolated systems tend toward equilibrium, open systems tend to maximize dissipated energy (Dewar, 2010) by self-organizing. Plant functions can be seen as a cascade of DS: fluctuations of stimuli are amplified by fluctuations of signaling molecules which in turn, are amplified by genes expression… which in the end, lead to the realization of the response function of the plant. A cascade of DS is intrinsically stochastic and the probability law is known.

As living systems could be interpreted as DS, they could exhibit the same four properties established by Per Bak. The next sections aim at showing the links between each properties of DS and biology of living organisms.

## 4. Amplification of fluctuations

A fluctuation is defined as a small variation of a physical quantity. If a system is in a metastable equilibrium state and if an input of energy is provided to it, it can tend toward a more stable equilibrium. This barrier of energy is called energy of activation and can be small enough to be provided by a fluctuation. Fluctuations at the molecular scale can create variation at the macroscopical scale. Fluctuations in the architecture of the cell polysaccharides suffice for changing the phyllotaxy of the plant which was modeled as a self-organizing process (Douady and Couder, 1996). For instance, fluctuations in the methylesterification status of cell wall pectin are amplified to modify the plant phyllotaxy (Peaucelle et al., 2008).

Oscillations are fluctuations, which occur around a mean value with a temporal pattern. In open systems, the dissipation of energy can generate spontaneous oscillations, which continue while energy is available (Kruse and Jülicher, 2005). The role played by oscillators in plant biology appears increasingly important. Genetic oscillators are involved in cell cycle (Tyson et al., 2001) and circadian clocks (Dunlap, 1999). They trigger cascades of DS allowing the plant adaptation to its environment as for instance, stress response (Mizuno and Yamashino, 2008) but also macroscopic traits such as the position of the lateral roots (Moreno-Risueno et al., 2010).

Fluctuations of cell wall mechanical stiffness give rise to expansive growth of pollen tubes (Zerzour et al., 2009; Rojas et al., 2011). These fluctuations produces cell growth, which can reach a 10^5^ size factor (Fayant et al., 2010).

## 5. Symmetry breaking

Anatomical and functional differences between the aerial part of the plant and its roots constitute the most obvious break of symmetry in plants. From the point of view of morphogenesis, the shape presenting the *most perfect symmetry* is a sphere, which is not the most common shape in nature. The shape of plant organs is determined locally by the rate and the direction of growth (Hamant and Traas, 2009). These parameters are determined by local mechanical properties such as cell wall stiffness (Guerriero et al., 2014) and local mechanical stresses exerted as example, by turgor pressure and/or surrounding cells. All these variables fluctuate in time and space which lead, with time, to the breaking of shapes symmetry.

The irreversibility of life involves breaks of temporal symmetry. The term *time's arrow* coined by Arthur Eddington is often used to emphasize the irreversible nature of time (Eddington, 1935). The direction of *time's arrow* is given by the rate of entropy. In biology, the debated notion of degree-day used to select plants at the same phase of development, is an adaptation of the notion of *time's arrow* (Bonhomme, 2000). This logical reasoning assumes that plants dissipate entropy proportionally to energy received. Under this hypothesis, degree-day is a coarse estimation of the thermodynamic time.

## 6. Creation and storage of information

Shannon (1948) established in the middle of the twentieth century, that entropy is oppositely linked to the amount of information (Stenholm, 2008). In open systems, entropy can decrease by exchange with the environment (|*dS_e_*| > 0 Figure 1B). The gain of information, corresponding to the reduction of entropy is consequently imported from the external environment. Information imported from the external environment can be stored. Memory is defined as the capacity of organisms to benefit from their past (Tulving, 1985). Plant have no organ dedicated to memorize information. Thus, plants have various kind of memory covering different characteristic times.

It can be as short time volatile memory. As an example, each solicitation of hairs of the Venus flytrap leaf hairs is stored as an accumulation of electrical energy (Volkov et al., 2008). If this electrical memory overcame a threshold during the 20 s following the first solicitation, the trap closes. The mechanism underlying the closure of the trap in ensured by a snap-buckling instability (Forterre et al., 2005). Proseus and Boyer (2008) demonstrated that an accumulation of free polysaccharides in the periplast of Chara corallina cells acts as a memory of approximately 1 h, storing low turgor pressure period. Barbacci et al. (2013) demonstrated that such mechanism can naturally be described by nonequilibrium thermodynamics. Epigenetic constitutes a reversible middle to long term memory, which stores information over several generations (Jaenisch and Bird, 2003).

Environmental information is stored and used to improve the adaptation of plant to its environment. For instance, water flux in root is regulated according to the water status history allowing the acclimation of the plant to water stress (Caldeira et al., 2014).

In the formulation of entropy provided by Shannon (1948), the notion of information is also linked to the notion of probability. Thus, living systems assimilated to DS exporting entropy are intrinsically stochastic which explains why experiments are often non-reproducible in biology.

## 7. Invariance to scale

As illustrated by previous examples, DS are not assigned to a special length scale. Thus, the concept of DS can be used to explore all aspects of biology (Gisiger, 2001). One consequence of the invariance to scale is the fractal-like organization of plant. This organization, apparently complex, leads to simple descriptive models called *allometric* models. Such allometric relations have been developed to model plant structures, such as the vascular system and to explain why energy use of plant is size independent (West et al., 2008).

## 8. Cascade of dissipative structure

A DS creates a cascade of DS through time and scale. The size of the cascade is variable meaning that the same causes may not produce the same observable effects. Indeed, Per Bak et al. (1988) demonstrated that the amplitude of energy dissipated by a DS is inversely proportional to the probability to observe it (the probability is noted f) which is modeled by an 1/f law. Hence, the higher the energy dissipated by the DS, the lower its frequency (Figure 1C). The 1/f law is invariant to scale and was, by consequence, used to describe processes at many scales from intracellular level (Plaxton, 1996) to ecosystems (Halley, 1996). Biological processes are intrinsically cascade of DS as for example metabolic pathways, such as glycolytic one (Plaxton, 1996). Plant shape regulation offers also good examples of cascade of DS. In special gravity-sensitive cells, statoliths in interaction with the cytoskeleton (Geitmann, 2006; Moulia and Fournier, 2009) self-organize to dissipate mechanical and thermal energy due to gravity and temperature. The self-organization consists in a complex movement of statoliths. For a long time, one considered that information on the actual position of the cell with respect to the vertical was perceived when all statoliths were sedimented and motionless at the bottom end of the cell. The present nonequilibrium thermodynamics framework teaches us that such configuration, close to the equilibrium, involves a little export of entropy and a little import of information. Raven and Rubery (1982) demonstrated that a memory (so an export of entropy) is mobilized to store information since the plant response seems to occur after signal averaging of integration. So to have a better understanding on the implication of statoliths in gravity perception, the most informative configuration to study is the transitive phase when more entropy is dissipated and information is collected. This phase corresponds to the stochastic movements of statoliths. Meroz and Bastien (2014) by deeper analysis, reached the same conclusion. Perception of gravity by statoliths constitutes the first DS of a cascade which ensures the gravitropic movement of the whole plant. Indeed, following gravity perception, transduction leads to the fluctuation of auxin content. The symmetry of auxin concentration is broken and increases in the lower side of the organ (Wisniewska et al., 2006). Increase in the concentration of auxin induces differential gene expression leading to cell elongation (which is a DS used to illustrate the previous point) and finally to the bending of the organ at the macroscopic scale (Bastien et al., 2013).

## Conclusion

Nonequilibrium irreversible thermodynamics offers a paradigm adapted to a holistic description of plant biology. From this point of view, biological processes are considered as DS. Each one of them creates a cascade of other DS in a *domino effect*. The probability for a DS to be created by cascade is oppositely linked to its amplitude and follows a global 1/f-law. The four features of a DS constitute the canvas to conduct analysis. Within this view, a biological process corresponds to amplifications of fluctuations. The key actors on which experiments and data analysis most focus, are those which break the symmetry of the initial configuration. Links with other biological processes or other plant functions are determined by exported entropy corresponding to gain of information and energy fluxes.

## Author contributions

AB, VM, ML Substantial contributions to the conception or design of the work; AB, VM, ML Drafting the work or revising it critically for important intellectual content; AB, VM, ML Final approval of the version to be published; AB, VM, ML Agreement to be accountable for all aspects of the work in ensuring that questions related to the accuracy or integrity of any part of the work are appropriately investigated and resolved.

## Funding

Pays de Loire regional project AIFruit, Impeto INRA project.

### Conflict of interest statement

The Associate Editor Yoël Forterre declares that, despite being affiliated with the same organization as the authors Adelin Barbacci and Vincent Magenet, the review process was handled objectively. The authors declare that the research was conducted in the absence of any commercial or financial relationships that could be construed as a potential conflict of interest.
